# Changes in geriatric rehabilitation: a national programme to improve quality of care. The Synergy and Innovation in Geriatric Rehabilitation study

**DOI:** 10.5334/ijic.2200

**Published:** 2015-12-15

**Authors:** Marije S. Holstege, Monique A.A. Caljouw, Ineke G. Zekveld, Romke van Balen, Aafke J. de Groot, Jolanda C.M. van Haastregt, Jos M.G.A. Schols, Cees M.P.M. Hertogh, Jacobijn Gussekloo, Wilco P. Achterberg

**Affiliations:** Department of Public Health and Primary Care, Leiden University Medical Center, Leiden, The Netherlands; Department of Research and Development, Evean, Purmerend, The Netherlands; Department of Public Health and Primary Care, Leiden University Medical Center, Leiden, The Netherlands; Department of Public Health and Primary Care, Leiden University Medical Center, Leiden, The Netherlands; Department of Public Health and Primary Care, Leiden University Medical Center, Leiden, The Netherlands; Department of General Practice and Elderly Care Medicine, VU University Medical Center, Amsterdam, The Netherlands; Department of Health Services Research, Maastricht University, Maastricht, The Netherlands; Department of Health Services Research, Maastricht University, Maastricht, The Netherlands; Department of Family Medicine, Maastricht University, Maastricht, The Netherlands; Department of General Practice and Elderly Care Medicine, VU University Medical Center, Amsterdam, The Netherlands; Department of Public Health and Primary Care, Leiden University Medical Center, Leiden, The Netherlands; Department of Public Health and Primary Care, Leiden University Medical Center, Leiden, The Netherlands

**Keywords:** geriatric rehabilitation, health service delivery, national programme, quality of care, care process

## Abstract

**Objective:**

To describe changes in the health service delivery process experienced by professionals, patients and informal caregivers during implementation of a national programme to improve quality of care of geriatric rehabilitation by improving integration of health service delivery processes.

**Study setting:**

Sixteen skilled nursing facilities.

**Study design:**

Prospective study, comparing three consecutive cohorts.

**Data collection:**

Professionals (elderly care physicians, physiotherapists and nursing staff) rated four domains of health service delivery at admission and at discharge of 1075 patients. In addition, these patients [median age 79 (Interquartile range 71–85) years, 63% females] and their informal caregivers rated their experiences on these domains 4 weeks after discharge.

**Principal findings:**

During the three consecutive cohorts, professionals reported improvement on the domain team cooperation, including assessment for intensive treatment and information transfer among professionals. Fewer improvements were reported within the domains alignment with patients’ needs, care coordination and care quality. Between the cohorts, according to patients (*n* = 521) and informal caregivers (*n* = 319) there were no changes in the four domains of health service delivery.

**Conclusion:**

This national programme resulted in small improvements in team cooperation as reported by the professionals. No effects were found on patients’ and informal caregivers’ perceptions of health service delivery.

## Introduction

In the Netherlands, postacute geriatric rehabilitation takes place in skilled nursing facilities, with a comprehensive rehabilitation team which often includes an elderly care physician, nursing staff, physiotherapist and occupational therapist, together with a psychomotor therapist, psychologist, social worker, speech therapist, dietician and pharmacist [1]. Geriatric rehabilitation is defined as a multidisciplinary set of evaluative, diagnostic and therapeutic interventions with the purpose to restore functioning or enhance residual functional capability in older people with disabling impairments [2]. The medical diagnosis for geriatric rehabilitation can be categorised into four main groups, i.e. stroke, trauma, joint replacement and a miscellaneous group for other diagnoses, i.e. chronic obstructive pulmonary disease (COPD), amputee, heart failure.

The organisation of geriatric rehabilitation is a complex care process, which suffers from a fragmented approach allowing room for improvement in the coordination, communication and continuity of care between the various health care providers and professionals involved [3]. Because most geriatric rehabilitation is provided after acute hospitalisation of older persons, effective collaboration between hospitals and postacute care settings for the development and performance of integrated care is essential [4–6]. Poor organisation of care has a negative impact on health care costs, patient outcomes and patient satisfaction with care [7,8].

An important challenge when developing integrated care is to get the patient and informal caregiver more involved in the rehabilitation process. Involvement of the patient and informal caregiver can improve continuity of care, quality of care and positive experiences with care [9–11]. Therefore, it is important to use different perspectives (patient/professional/informal caregiver) in the evaluation of processes and outcomes on the level of health service delivery (i.e. alignment with patients’ care needs; care coordination; team cooperation; and quality of care) [12]. However, few studies have evaluated multiple perspectives involved with changes in health service delivery [9,10,13].

To improve the quality of service delivery for geriatric rehabilitation in the Netherlands, a national programme was initiated by the Dutch Ministry of Health, Welfare and Sport in 2011. The aim of this programme was to stimulate self-organising capacity to develop integrated geriatric rehabilitation in order to improve the health service delivery. This programme was introduced at a time when the health care system was transforming from a typical long-term care government-guided reimbursement system without financial incentive for efficient geriatric rehabilitation, towards a more market-guided bundled payment system. Internationally, bundled payment systems appear to be a strong incentive for collaborating geriatric rehabilitation service organisations with the goal to improve quality of care [6,13].

The aim of this study is to describe changes in the health service delivery process during implementation of the national programme, as experienced and rated by professionals, patients and their informal caregivers.

## Methods

### Study design

During implementation of the national programme in the Netherlands, a prospective longitudinal mixed method study was performed among the participating skilled nursing facilities, i.e. the Synergy and Innovation in Geriatric Rehabilitation (SINGER) Study. For data collection, three consecutive cohorts (each cohort recruited over a 4-month period in March 2011, September 2011 and March 2012) were used to evaluate changes in perceptions of health service delivery during implementation of the national programme. The first cohort was enrolled at the start of the implementation phase, and the second and third cohorts at 6 and 12 months, respectively, after the start of the national programme. The process evaluation with quantitative measures was postulated by the Dutch Ministry of Health.

### Participants

Eighty groups of collaborating geriatric rehabilitation service organisations that wanted to participate in the national programme provided an action plan outlining the goals they aimed to achieve to improve their quality of care. A geriatric rehabilitation service organisation consisted of at least one skilled nursing facility, a hospital and a health insurance company, but could also include home care providers, primary care providers (e.g. physiotherapists, occupational therapists) or rehabilitation centres. From the 80 available geriatric rehabilitation service organisations, the Dutch Ministry of Health, Welfare and Sport selected 16 for the national programme based on their initial plans and national coverage. Data collection took place in the skilled nursing facilities of the selected organisations.

Patients admitted to a participating skilled nursing facility for geriatric rehabilitation were recruited for participation. For each participating patient, their professional caregivers (elderly care physician, physiotherapist and one delegate of the nursing staff) and informal caregivers involved were also invited to participate. The study population was recruited in three consecutive cohorts starting in March 2011, September 2011 and March 2012 (spanning a 4-month period for each cohort).

Each skilled nursing facility was asked to include a minimum of (the first) 10–15 admitted patients, stratified for diagnostic group, in each cohort. Excluded from the study were patients with a diagnosis of dementia.

A waiver of consent was given by the Medical Ethics Committee of the Leiden University Medical Center (LUMC).

### National intervention programme

The Ministry of Health, Welfare and Sport initiated the national programme with the aim to stimulate self-organising capacity to develop integrated geriatric rehabilitation in order to improve the health service delivery. The ministry provided financial support to the participating geriatric rehabilitation service organisations for internal project management. The health insurance companies provided financial incentives for the more intensive treatment programmes. In addition, the geriatric rehabilitation service organisations themselves contributed to the implementation of their goals to improve geriatric rehabilitation service delivery. Each participating geriatric rehabilitation service organisation was responsible for the internal project organisation, implementation of their action plan and for achievement of their formulated goals. During implementation of the programme, nine national meetings were held with the project board and members of the participating geriatric rehabilitation service organisations. The project board consisted of an expert team of stakeholders with the aim to initiate, identify and disseminate best practices between the involved geriatric rehabilitation service organisations. During these meetings, representatives of the 16 geriatric rehabilitation service organisations shared their experiences and knowledge. In addition, preliminary process outcomes on this national evaluation study were presented as feedback for the ongoing implementation process. To monitor and supervise the action plans and goals, using the plan-do-study-act cycles [14], two national process managers visited the internal project managers of each geriatric rehabilitation service organisations at the start of the implementation (between July and December 2011) and twice during follow-up. These process managers had a more qualitative approach and interviewed the internal project managers of each geriatric rehabilitation service organisations on the facilitators and barriers of this national incentive and reported in a process evaluation [15]. The lessons learned from these interviews were reported in a guidebook [16] and summarised in Box 1.

### Content of the programme

To improve the geriatric rehabilitation service, each geriatric rehabilitation service organisation set its goals to optimise integrated care. Integrated care is defined as ‘*a concept bringing together inputs, delivery, management and organization of services related to diagnosis, treatment, care, rehabilitation, and health promotion. Integration means to improve the service in relation to access to care, quality of care, user satisfaction, and efficiency of care’* [17]. For that purpose each geriatric rehabilitation service organisation developed or improved care pathways for a specific group, i.e. stroke, joint replacement and hip fracture, as well as for other smaller groups of specific diseases (i.e. COPD, amputation, heart failure), or for all patient groups. A care pathway is defined as a complex intervention for the mutual decision-making and organisation of care processes for a well-defined group of patients during a well-defined period [18,19].

Within the national programme the main goals of development of integrated care in geriatric rehabilitation can be divided into four domains of (geriatric rehabilitation) service delivery, according to the evaluation model of Hartgerink et al. [12], i.e. (1) with patients’ (care) needs; (2) care coordination; (3) team cooperation; and (4) quality of care. Box 2 presents the main goals of development in this national programme based on these domains and aiming to improve quality of care.

### Data collection and outcome measures

Professional caregivers collected patient characteristics, i.e. age, gender, Barthel Index [20] and indication for geriatric rehabilitation by diagnostic group, as well as process outcomes of geriatric rehabilitation service delivery, were collected for each cohort at admission and again at discharge by means of an online questionnaire. A helpdesk was available for any questions concerning the online questionnaire.

In addition, patients and informal caregivers filled in a (paper version) questionnaire to measure their experiences with the process of geriatric rehabilitation service delivery 4 weeks after patient discharge.

The experience with the geriatric rehabilitation health service delivery processes was measured with self-developed questionnaires based on face validity for the professionals, as well as for patients and informal caregivers; all questions (answered on a 4-point Likert scale) concerned the four domains of health service delivery. Questions on (1) alignment with patients’ care needs were filled out by the elderly care physicians and physiotherapists; on (2) care coordination were filled out by the elderly care physicians and a member of the nursing staff; on (3) team cooperation were filled out by all three professionals; and questions on (4) care quality were filled out by the nursing staff.

The questionnaire for the patients and informal caregivers covered also all these four domains.

### Statistical analysis

Descriptive statistics were used to analyse outcomes on the four domains of health service delivery as reported by the professionals, patients and informal caregivers. For each question, the percentage of the category ‘good and excellent’ was reported versus the answer option ‘poor and fair’. To compare the outcomes of the three consecutive cohorts, *P* for trend values were calculated with the Kruskal–Wallis test, and, in case of numeric data, values were calculated with one-way analysis of variance (ANOVA). A *P* for trend ≤ 0.05 was considered statistically significant. All patients with data from all three professional caregivers at admission to the skilled nursing facility (baseline) and who had not died and who were not readmitted to hospital during the rehabilitation stay were included for analysis of the process outcomes at admission, discharge and four weeks’ follow-up.

Data were analysed using IBM SPSS Statistics for Windows, version 20.0.

## Results

### Response and background characteristics

**Figure 1.**
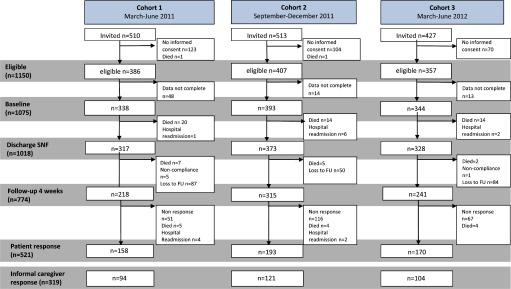
Flowchart of patient recruitment and follow-up in the 16 skilled nursing facility. Analysed data include data rated by professional caregivers at admission and discharge, and response at 4 weeks’ follow-up, of patients and their informal caregivers.

The flowchart of patient recruitment and follow-up is presented in Figure 1.

Of the 1150 eligible patients, at baseline 1075 patients (93.5%) had completed questionnaires from all three professionals and were included in the present study. At discharge 1018 patients (95%) were included for data analysis. Of this latter group, at 4 weeks after discharge 774 patients were eligible for follow-up measurement. Finally, 512 patients and 319 of their informal caregivers had provided a response to the follow-up questionnaire.

Each of the 16 skilled nursing facilities included a median of 46.5 (IQR 28–126) patients. Overall, the baseline population of patients (*n* = 1075) had a median age of 79 (IQR 71–85) years, consisted of 63% females, and were categorised into stroke (36%), elective joint replacement (15%), traumatic injuries (25%), and other smaller groups of specific diseases (i.e. COPD, amputation, heart failure (24%)). There were no differences in age, gender and baseline Barthel Index between the cohorts. The informal caregivers (*n* = 319) had a median age of 65 (IQR 56–75) years and consisted of 66% females. The relationship between informal caregivers and patients was: spouse (49%), sibling (4%), daughter or son (36%) and other relation (11%). There were no differences in age, gender and type of relationship between the cohorts.

### Process outcomes

Tables 1–4 present the outcomes (in percentage ‘good and excellent’) on geriatric rehabilitation service delivery process as reported by the professionals (elderly care physicians, nursing staff and physiotherapists), patients and their informal caregivers.

### Alignment with patients (care) needs: do professionals give what patients need? (Table 1)

#### Professionals

Involvement of the patient by the physiotherapist in setting rehabilitation goals decreased across three cohorts (*P* trend = 0.05). Elderly care physicians reported high patient involvement in setting rehabilitation goals in all cohorts, with no significant change between the cohorts (*P* trend = 0.69). In contrast, the percentage involvement of the informal caregiver in setting rehabilitation goals had increased (*P* trend < 0.01), as reported by elderly care physicians. Physiotherapists reported that in total (all three cohorts together) 155 (21%) of the informal caregivers were involved in setting rehabilitation goals, but with no change over time (*P* = 0.85).

Across three cohorts, there was an increase in the percentage of patients and/or informal caregivers attending the multidisciplinary team (meeting or the discussion of individual care plans, as reported by elderly care physicians (*P* trend = 0.05)).

#### Patients and informal caregivers

Across three cohorts there was a non significant increase in the percentage involvement of setting rehabilitation goals, as reported by the informal caregivers (*P* -trend: 0.06). In total, 312 (61%) patients and 150 (48%) informal caregivers reported a ‘good’ or ‘excellent’ way of dealing with individual needs, with no difference between the three cohorts (*P* trend = 0.85 and 0.74, respectively).

In total, 48% of the patients and 52% of the informal caregivers were involved in the decision-making process for referral to a rehabilitation location after a hospital stay, with no difference in trend between the cohorts (*P* trend = 0.38 and 0.85, respectively).

### Care coordination (Table 2)

#### Professionals

Across three cohorts, professionals gave a higher rating (percentage ‘good or excellent’) for guidance and support of patients’ transfer from hospital to a skilled nursing facility (*P* trend < 0.01). The rating of patients and informal caregivers for guidance and support with the transfer from a skilled nursing facility to home remained the same in all three cohorts (*P* = 0.96 and *P* = 0.84, respectively), as did the rating for the preparation of the patient for discharge home (overall 91%, *P* trend = 0.84).

#### Patients and informal caregivers

The rating for guidance and support with the transfer from hospital to skilled nursing facility (percentage ‘good’ or ‘excellent’) did not change over time, as rated by patients (*P* trend = 0.50) and informal caregivers (*P* trend = 0.38); neither did satisfaction with the transfer from skilled nursing facility to home as reported by patients (*P* trend = 0.42) and informal caregivers (*P* trend = 0.54).

### Team cooperation (Table 3)

#### Professionals

There was an improvement in the rating (percentage ‘good or excellent’) of the information handover between professionals from hospital to skilled nursing facility, as reported by the nursing staff (*P* trend ≤ 0.01) and elderly care physicians (*P* trend = 0.04). Rating of the information handover between physiotherapists improved significantly from skilled nursing facility to follow-up care (*P* trend = 0.01) and did not change between nurses and between medical specialists from skilled nursing facility to follow-up care.

There was an increase of the (small) percentage of elderly care physicians who participated in the MDT hospital meetings (*P* trend = 0.04), to determine the priority of need and proper place of treatment (triage). There was no change in the percentage of consultations by rehabilitation physicians during the rehabilitation stay (*P* trend = 0.14).

In the skilled nursing facility, in 98% of the MDT meetings the team consisted of an elderly care physician, a physiotherapist and a member of the nursing staff. In addition, the multidisciplinary team (MDT) meetings consisted of an occupational therapist (79%), speech therapist (39%), a dietician (26%) and other professional(s) (51%) (i.e. psychologist, social worker, creative therapist, nurse practitioner, case manager). Only participation of the occupational therapist showed an increase across cohorts (*P* trend < 0.01). Rehabilitation goals were evaluated weekly or every two weeks for 64% of the included patients.

According to the elderly care physician, the amount of patients assessed for the indication of more intensive treatment at the rehabilitation ward increased by 10% between cohort 1 and cohort 3 (*P* trend = 0.01).

#### Patients and informal caregivers

In all cohorts, patients and informal caregivers reported similar percentages for good and excellent alignment of the professionals.

### Quality of Care (Table 4)

#### Professionals

According to the nursing staff, patients (*P* trend = 0.03) and informal caregivers (*P* trend = 0.51) received sufficient information about care and treatment during rehabilitation.

The percentage of patients receiving more (or more intensive) treatment (≥ 4 hours/week) increased, as reported by the elderly care physicians (*P* trend < 0.01).

Only longer treatment periods (i.e. more treatment time during each session) decreased from 11% in cohort 1 to 2% in cohort 3 (*P* trend < 0.01). The amount of group therapy increased between the cohorts from 13% in cohort 1 to 30% in cohort 3 (*P* trend < 0.01). According to the physical therapists, a low percentage of patients (overall 13.9%: *P* trend = 0.71) performed individual exercise without the supervision of a physical therapist; in contrast, the nursing staff reported that 68% of the patients performed daily individual exercise. Also, there was more physical activity at the rehabilitation ward under the supervision of the nursing staff (*P* trend = 0.01).

#### Patients and informal caregivers

Overall, patients and informal caregivers rated the total care pathway as 7.3 (SD 1.3) on a 0–10 scale (with 10 indicating excellent). The level of satisfaction did not differ between the cohorts. In total, 390 (77%) patients and 201 (67%) informal caregivers rated the care and treatment during rehabilitation stay as good or excellent.

In total, 286 (60%) patients and 142 (51%) informal caregivers reported the received information from professionals to be ‘good’ or ‘excellent’. Also, 415 (88%) patients and 268 (92%) informal caregivers reported that the patient was referred in a proper manner from hospital to skilled nursing facility for rehabilitation, with no change over the cohorts.

Overall 71% (*n* = 364) of the patients and 78% (*n* = 243) of the informal caregivers reported that there was enough (or more than enough) possibility to perform individual exercise at the rehabilitation ward without supervision of a physical therapist; this did not differ between the cohorts.

## Discussion

This study evaluated the perceptions of professionals, patients and informal caregivers related to the quality of health service delivery in geriatric rehabilitation during implementation of a national programme aimed at improving quality of geriatric rehabilitation in the Netherlands. The study underlines that geriatric rehabilitation is a multidisciplinary process aiming to achieve integrated patient-centred care [3].

Professionals reported small but positive effects on several items of health service delivery, mainly on the domain team cooperation. Within the domains alignment with patients needs, care coordination and care quality, less changes were reported. In cohort 1, the perception of the quality of the service delivery was already high, indicating that professionals were largely satisfied with the service they provided. Our results also show positive patient and informal caregiver perceptions on the quality of geriatric rehabilitation service delivery. The level of satisfaction of patients and informal caregivers did not change during implementation of the programme. An explanation for this may be that patient satisfaction is related to service delivery and is based on expectations and personal interactions, rather than on the quality of technical competence [21].

Our results are in line with the national integrated care pilot in the UK [13] in which improvements appeared on a process level, but had limited effects on patient satisfaction. However, after implementation of quality improvements, a longer period of evaluation may be needed to reveal changes in service delivery as experienced by patients and informal caregivers [9]. It is a worldwide challenge to initiate, develop and evaluate integrated care on a large scale with multiple health care providers involved in a changing health care economy, also called ‘complex adaptive systems’ or ‘complex interventions’ [22–24]. These systems are complex because of the dynamics within the different health care providers and the large number of components that interact when developing integrated care delivery [13,22,23,25,26]. Another explanation may be that the national project had too optimistic expectations about the capacity of the organisations to execute a successful change themselves. Although there was central monitoring of the goals and progress next to exchange of experiences between organisations, little was done on education and coaching of effective ways of change management in these complicated integrated care processes.

### Strengths and limitations

One strength of this study is the use of multiple data sources, including the patient, informal caregiver and three core professionals (elderly care physicians, physiotherapists and nursing staff) to gain a broad perspective on the perceptions of health care delivery in skilled nursing facilities. Also, the study has a high response rate from the professionals.

The present study can be seen as having a type of active participatory research design. To achieve good adaptation in a real-world setting, an active research design has several advantages [13,22]. Development, implementation and evaluation were combined to develop tailor-made integrated care. The developments covered the different aspects of health care delivery and all stakeholders were committed to improve the quality of care. The collaboration between hospitals, skilled nursing facilities, homecare, health insurance companies and the government resulted in a process to innovate and exchange knowledge. This national programme stimulated the self-organising capacity of the participants and resulted in a national movement of development in skilled nursing facilities.

The study also has some limitations. First, the process outcomes of the professionals were based on self-rating, which may have led to more social desirable answers. However, quality outcomes were also based on rating by patients and informal caregivers, who were not aware of the changes. Second is that the ratings of the process and outcomes of professionals, patients and informal caregivers were already high at baseline, leaving little room for improvement (ceiling effect). Third is that the Dutch Ministry of Health may have selected relatively good quality geriatric rehabilitation service organisations, whereas a selection based on relatively poor performance by means of quality indicators might leave more room for improvement. Finally, within this study we were particularly interested to explore the changes in the health service delivery process experienced by professionals, patients and informal caregivers. Other factors depending on organisational characteristics of the skilled nursing facilities would be of interest for further research, since these characteristics could influence the expected level of change as well [12]. However, this was outside the scope of our study.

This study reports on a national programme to improve integrated care in geriatric rehabilitation. Professionals, informal caregivers and patients reported some and small improvements in the care process. Effective change in complex integrated care processes and the measurement of the effects on process outcomes remains a challenge.

## Conclusion

This national programme to improve quality of care in geriatric rehabilitation resulted in small improvements in team cooperation, as reported by the professionals. However, no effects were found for patients’ and informal caregivers’ perceptions on health service delivery. These results may suggest that changes in organisational structure need time to penetrate to the outcome level of patients and informal caregivers

## Figures and Tables

**Box 1.**
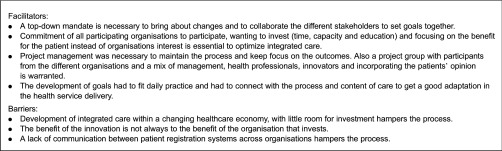
Facilitators and Barriers during the national incentive according to the national process managers

**Box 2.**
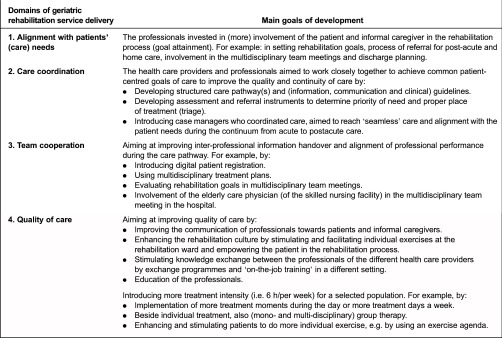
Main goals of development of integrated care within the skilled nursing facilities in the four domains of geriatric rehabilitation service delivery

**Table 1.**
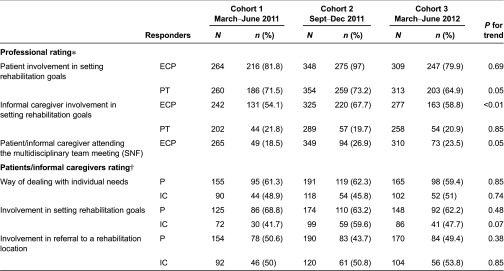
*Alignment with patients’ (care) needs, do professionals give what patients need?* According to professionals (elderly care physicians and physiotherapists), patients and informal caregivers in percentage good and excellent. Selection of patients with completed professional data at baseline who had not died and were not readmitted to hospital during their rehabilitation stay (*n* = 1018)

ECP, Elderly care physician; PT, physiotherapist; P, patient; IC, informal caregiver. Values are numbers (% good and excellent) unless indicated otherwise. *P* for trend values were calculated with the Kruskal–Wallis test.

Professional rating *N* total: C1: *n* = 317; C2: *n* = 373; C3: *n* = 328.

Patient rating *N* total: C1:158; C2:193; C3:170.

Informal caregiver rating *N* total: C1:94; C2:121; C3:104.

*Rated at discharge from skilled nursing facility (SNF).

†Rated 4 weeks after discharge SNF.

**Table 2.**
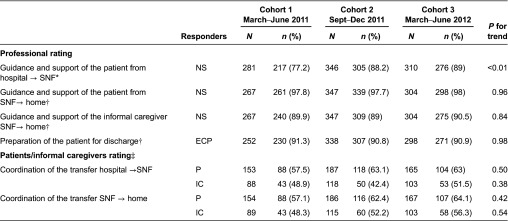
*Care coordination* according to professionals (elderly care physicians and nursing staff), patients and informal caregivers in percentage good and excellent. Selection of patients with completed professional data at baseline and not-died or having a readmission to hospital during their rehabilitation stay (*n* = 1018)

NS, Nursing staff; ECP, elderly care physician; P, patient; IC, informal caregiver. Values are numbers (% good and excellent) unless indicated otherwise. *P* for trend values were calculated with the Kruskal–Wallis test.

Professional rating *N* total: C1: *n* = 317; C2: *n* = 373; C3: *n* = 328.

Patient rating *N* total: C1:158; C2:193; C3:170.

Informal caregiver rating *N* total: C1:94; C2:121; C3:104.

*Rated at admission skilled nursing facility (SNF).

†Rated at discharge SNF.

‡Rated 4 weeks after discharge SNF.

**Table 3.**
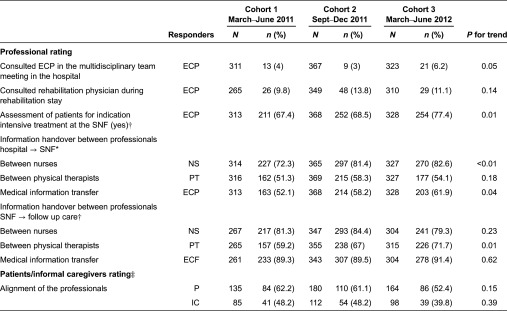
*Team cooperation* according to professionals (elderly care physicians, nursing staff and physical therapists), patients and informal caregivers in percentage good and excellent. Selection of patients with completed professional data at baseline and not-died or having a readmission to hospital during their rehabilitation stay (*n* = 1018)

NS, Nursing staff; ECP, elderly care physician; PT, physical therapist; P, patient; IC, informal caregiver. Values are numbers (% good and excellent) unless indicated otherwise. *P* for trend values were calculated with the Kruskal–Wallis test.

*Rated at admission skilled nursing facility (SNF).

†Rated at discharge SNF.

‡Rated 4 weeks after discharge SNF.

Professional rating *N* total: C1: *n* = 317; C2: *n* = 373; C3: *n* = 328.

Patient rating *N* total: C1:158; C2:193; C3:170.

Informal caregiver rating *N* total: C1:94; C2:121; C3:104.

**Table 4.**
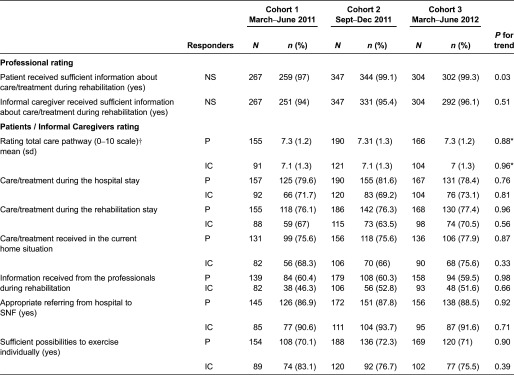
*Care quality* at discharge skilled nursing facility (SNF) according to nursing staff and at 4 weeks’ follow-up according to patients and informal caregivers in percentage good and excellent. Selection of patients with completed professional data at baseline and not-died or having a readmission to hospital during their rehabilitation stay (*n* = 1018)

SNF, Skilled nursing facility; NS, nursing staff; P, patient; IC, informal caregiver. Values are numbers (% good and excellent) unless indicated otherwise. *P* for trend values calculated with the Kruskal–Wallis test.

Professional rating *N* total: C1: *n* = 317; C2: *n* = 373; C3: *n* = 328.

Patient rating *N* total: C1:158; C2:193; C3:170.

Informal caregiver rating *N* total: C1:94; C2:121; C3:104.

*One-way ANOVA.

†10 indicating excellent.
